# Electrical Stimulation in Bone Healing: Critical Analysis by Evaluating Levels of Evidence

**Published:** 2011-07-26

**Authors:** Michelle Griffin, Ardeshir Bayat

**Affiliations:** ^a^Department of Plastic and Reconstructive Surgery Research, School of Translational Medicine, Manchester Interdisciplinary Biocentre, University of Manchester, United Kingdom; ^b^Department of Plastic and Reconstructive Surgery, University Hospital of South Manchester NHS Foundation Trust, Wythenshawe Hospital, Southmoor Road, Manchester, United Kingdom; ^c^University of Manchester, Manchester Academic Health Science Centre, University Hospital of South Manchester NHS Foundation Trust, Wythenshawe Hospital, Southmoor Road, Manchester, United Kingdom

## Abstract

**Objectives:** Direct current, capacitive coupling, and inductive coupling are modes of electrical stimulation (ES) used to enhance bone healing. It is important to assess the effectiveness of ES for bone healing to ensure optimization for clinical practice. This review aims to examine the level of evidence (LOE) for the application of ES to enhance bone healing and investigate the proposed mechanism for its stimulatory effect. **Methods:** MEDLINE and EMBASE searches were conducted to identify clinical and in vitro studies utilizing ES for bone healing since 1959. A total of 105 clinical studies and 35 in vitro studies were evaluated. Clinical studies were assigned LOE according to Oxford Centre for Evidence Based Medicine (LOE-1, highest; LOE-5, lowest). **Results:** Direct current was found to be effective in enhancing bone healing in spinal fusion but only LOE-4 supported its use for nonunions. Eleven studies were retrieved for capacitive coupling with LOE-1 demonstrating its effectiveness for treating nonunions. The majority of studies utilized inductive coupling with LOE-1 supporting its application for healing osteotomies and nonunions. In vitro studies demonstrate that ES enhances bone healing by changes in growth factors and transmembrane signaling although no clear mechanism has been defined. **Conclusion:** Overall, the studies, although in favor of ES application in bone repair, displayed variability in treatment regime, primary outcome measures, follow-up times, and study design, making critical evaluation and assessment difficult. Electrical stimulation shows promise in enhancement of bone healing; however, better-designed clinical studies will enable the optimization for clinical practice.

When bone encounters injury, it undergoes a unique process of self-regeneration to form new bone to heal itself. However, in 5% to 10% of patients this process is disrupted which leads to delayed bony healing or nonunions.^1^ This is of great consequence to the clinician as nonunions pose a huge burden on the individual in terms of continuing pain and disruption to their daily activities and increases the expenditure of medical resources. Therefore, finding effective methods to enhance bone healing has been of great research interest, one of which is the use of electrical stimulation (ES).

In the early 1950s, Fukada and Yasuda^2^ demonstrated that when stress is applied to bone in such a way to cause deformity electrical potentials are generated, in areas of compression the bone was electronegative and caused bone resorption, whereas areas under tension were electropositive and produced bone. Therefore, subsequent developments were based on the idea that stimulating these endogenous electric fields using an ES device would enhance bone healing.^3^

There are 3 methods of administering electrical current to bone (Fig 1), which have been used in clinical practice (Table 1) including direct current (DC), capacitive coupling (CC), and inductive coupling (IC). In several models, DC involves invasive surgical placement of electrodes.^1^ A cathode is placed at the site of the bone defect with an anode in the soft tissue nearby.^3^ Osteogenesis has been shown to be stimulated at the cathode using currents between 5 and 100 µA and varying the number of electrodes between 2 and 4.^3^ Since the stimulator is implanted, the therapeutic treatment is continuous but is removed once healing has occurred. Direct current is advantageous as patient compliance is minimal; however, the technique is invasive with risk of infection, tissue reaction, and soft tissue discomfort.^4^

Capacitive coupling involves noninvasive placement of 2 cutaneous electrodes on opposite sides of the bone to be stimulated.^3^ A power source, usually attached to the patients cast is then connected to the electrodes forming an electrical field within the fracture site. Using potentials of 1 to 10 V at frequencies between 20 and 200 kHz creates electric fields of 1 to 100 mV/cm, which has shown to be efficient for bone stimulation.^5^

Inductive coupling enhances bone healing by using pulsed electromagnetic field (PEMF) stimulation. Inductive coupling is formed by placing 1 or 2 current-carrying coils on the skin over the fracture site.^4^ As current flows through the coils, an electromagnetic field radiates at right angles to the coil base but within the fractures site.^4^ The electrical field that is formed varies in size because of the type of tissues at the fracture site and the properties of the applied magnetic field.^5^ Electromagnetic fields varying from 0.1 to 20 G have been used to create an electrical field at the fracture site of 1 to 100 mV/cm.^6^ Inductive coupling and CC are beneficial treatment options for patients as they are noninvasive, painless, and surgery free.^4^ Furthermore, they can be easily and conveniently used by patients at home and in most cases patients are allowed to bear weight.^4^

Electrical stimulation has shown to be effective in aiding bone healing in a variety of orthopedic conditions such as aiding internal and external fixation,^7^ enhancing delayed or nonunion fractures^8^ and osteotomies,^9^ improving the efficacy of bone grafts,^10^ treating fresh fractures,^11^ and aiding femoral osteonecrosis.^12^ However, the mechanism by which ES has its stimulatory effect in enhancing bone healing remains unclear.^3^

Therefore, we performed a systematic review to address (1) what is the proposed mechanism of action for DC, IC, and CC (2) what is the level of evidence (LOE) supporting the use of DC, CC, and IC in enhancing bone healing for orthopedic conditions.

## MATERIALS AND METHODS

An electronic search of the MEDLINE through PubMed and EMBASE databases was performed to identity all relevant clinical studies that utilized ES for the treatment of bone healing from 1959 to 2009 by 2 independent reviewers (M.G., A.B.). Over the same time period, all in vitro studies that assessed the mechanism behind ES were identified. Keywords with Boolean operators used in the search included the following: “bone healing” or “nonunion” or “fracture healing” or “fracture ununited” and ES or electrical therapy or electromagnetic field stimulation or pulsed electromagnetic field stimulation. Articles were considered eligible if included the following inclusion criteria: (1) inclusion of a treatment arm receiving ES of DC, CC, or PEMF to impact bone healing; (2) evaluated the use of ES treatment for long bone and non–long bone healing (spine, scaphoid, and clavicle); (3) evaluated the use of ES to impact bone healing including the effect of ES on enhancing nonunion or malunion or delayed union, spinal fusion, pseudoarthrosis, osteotomies, fresh fractures, and femoral osteonecrosis; and (4) in vitro studies that evaluated the mechanism behind DC, CC, or PEMF. Case reports and expert opinions were included to that all related studies were identified and reviewed. Articles were excluded if they were (1) not published in English, as the reviewers would not fully understand the manuscript; (2) animal studies as these reports only show the end result whether there has been an increase or decrease in bone development and do not give details for the mechanism of ES; (3) mode of ES other than DC, CC, or IC; and (4) review papers, editorials, publications on congress meetings, unpublished data, or letters to the editor. Review articles were only used to identify any other relevant articles.

Clinical studies were then grouped by the primary method of ES used (DC, CC, or IC) and then assessed and assigned an LOE adapted from the Oxford Centre for Evidence Based Medicine (http://www.cebm.net/index.aspx?o=1025) to establish whether valid and reliable evidence supports the use of ES for bone healing. These levels, ranging from LOE-1 to LOE-5, are based on methodology and study design. In brief, these were how LOEs were assigned as follows: LOE 1 = randomized control trial; LOE-2 = cohort study; LOE-3 = case-control study; LOE-4 = Case series study; LOE-5 = expert opinion or case report.

The clinical studies were further evaluated for their study design and assessed for the direction of the main conclusion regarding the efficacy of the ES method used. To aid to this process, the following data were extracted from the clinical studies: (1) primary outcome measure, (2) assessment time—time over which ES was monitored, (3) ES treatment regime, (4) main findings, and (5) main conclusion drawn by the authors. A grade of recommendation was then assigned according to Oxford Centre for Evidence Based Medicine guidelines based on the findings for each mode of ES for different clinical situations (Table 2). In brief, the criteria used was as follows:

**Table:** 

A = consistent level 1 studies
B = consistent level 2 or 3 studies *or* extrapolations* from level 1 studies
C = level 4 studies *or* extrapolations from level 2 or 3 studies
D = level 5 evidence *or* troublingly inconsistent or inconclusive studies of any level

*“Extrapolations” are where data is used in a situation that has potentially clinically important differences than the original study situation.

**Figure 2:**
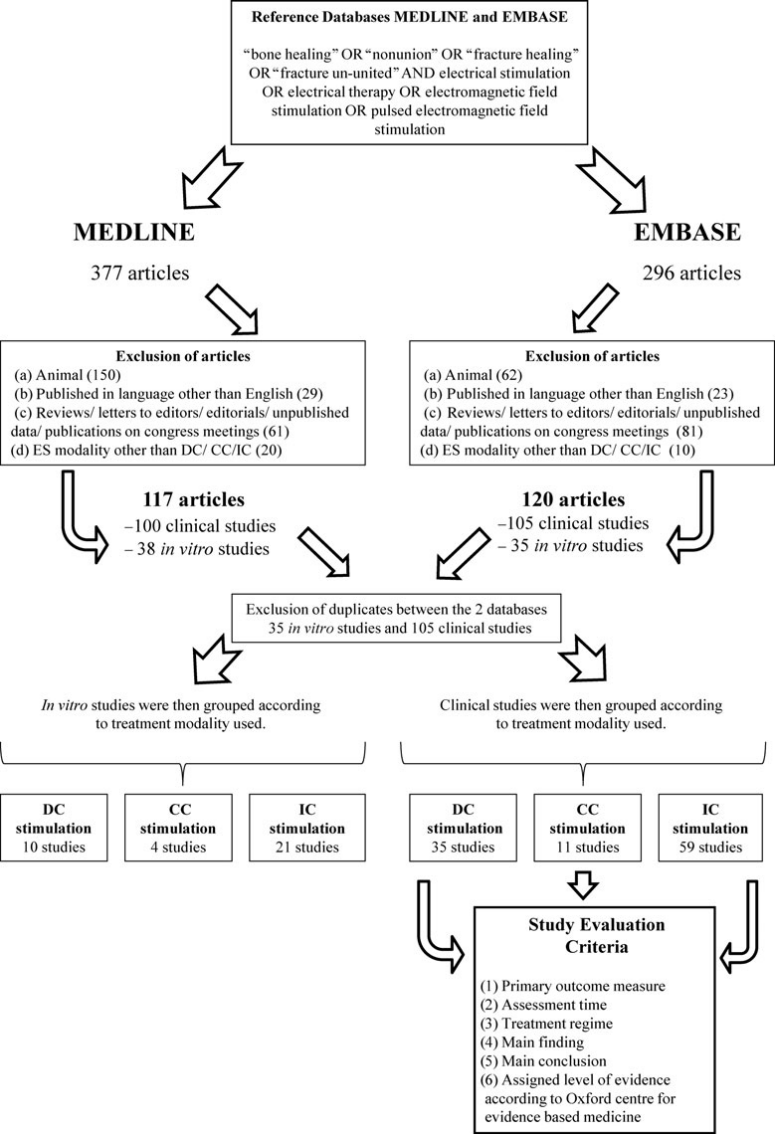
Flow chart demonstrates the selection criteria and process employed in the study. CC indicates capacitive coupling; DC, direct current; ES, electrical stimulation; IC, inductive coupling.

Figure 2 shows an overview of the selection of the studies used and the final articles selected for each type of ES for both clinical and in vitro studies.

## RESULTS

### Mechanism of action of ES

**Figure 3:**
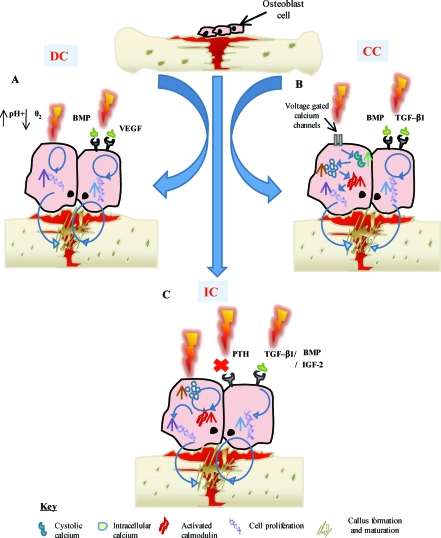
The proposed mechanism of action of the different types of electrical stimulation methods. (*a*) Proposed mechanism for direct current (DC). Direct current lowers the oxygen level and increases the pH, which causes an increase in osteoblast cell proliferation. This in turn enhances callus formation and maturation, leading to bone healing. All 3 types of ES enhance growth factors. This in turn increase cell proliferation, which enhances callus formation and maturation, leading to bone healing and improved clinical outcome. (*b*) Proposed mechanism for capacitive coupling (CC). Capacitive coupling causes an increase in cystolic calcium through voltage gated calcium channels. This then increases intracellular calcium, which in turn enhances activated calmodulin stores. Cell proliferation then increases, which enhances callus formation and maturation, leading to bone healing. (*c*) Proposed mechanism for inductive coupling (IC). Inductive coupling causes a direct increase in intracellular calcium, which in turn enhances activated calmodulin stores. Cell proliferation is increased, which enhances callus formation and maturation, leading to bone healing. BMP indicates bone morphogenetic protein; IGF-2, insulin growth factor 2; PTH, parathyroid hormone; TGF-β1, transforming growth factor beta 1; VEGF, vascular endothelial growth factor.

The in vitro studies evaluated report that DC stimulates osteogenesis by an electrochemical reaction at the cathode (O_2_ + 2H_2_O + 4e− → 4OH) creating end products referred to as faradic products.^13^-^22^ The production of hydroxyl ions (OH) at the cathode are shown to lower the oxygen concentration and increase the pH.^15^ This environment prevents bone resorption and increase bone formation by increasing osteoblast and decreasing osteoclast action.^15^ A second faradic product hydrogen peroxide (H_2_O_2_) is also formed at the cathode,^15^ which enhances osteoclast differentiation.^20^ The resorption by the osteoclasts in turn triggers bone formation by the osteoblasts. The effect of H_2_O_2_ could also be due to its stimulatory action on vascular endothelial growth factor secretion by macrophages, which is important for angiogenesis in fracture healing.^18^ Evidence also shows that DCs' stimulatory effect may be due to an increase in growth factor synthesis by osteoblasts, in particular bone morphogenetic protein (BMP)-2,6,7.^19^ Figure 3a shows a summary of DC-proposed mechanism of action.

The vitro studies reviewed^23^^-^26 that use CC describe the main mechanism by which CC stimulates bone formation is by calcium translocation via voltage-gated calcium channels.^23^^,^^24^ This mechanism was proved when verapamil was administered to block the Ca^2+^ channels in osteoblasts treated with CC, as the cell proliferation consequently decreased.^24^ However, once the calcium voltage-gated channels are activated, this triggers an augmenting pathway. First, there is an increase in phospholipase A_2_, which raises prostaglandin E_2_ synthesis.^23^ This then amplifies cystolic Ca^2+^, which increases intracellular calcium stores to activate the last step in the pathway, of enhancing the activated calmodulin levels.^23^ Activated calmodulin has been shown to promote cellular proliferation in bone by upregulating nucleotide synthesis and a wide array of enzymatic proteins, which enhances callus formation and maturation.^23^ Studies also report that CC enhances bone healing by the activation of growth factors, for example, mRNA expression of BMP-2,4,5,6,7^25^ and transforming growth factor-beta 1 (TGF-β1) is increased by osteoblasts stimulated by CC.^26^ Figure 3b shows a summary of CC-proposed mechanism of action.

Two mechanisms are described by which IC has its stimulatory effect.^23^^,^^27^^-^47 First, IC exhibits its effect on bone healing by increasing the calcium uptake of bone. This is achieved by inactivating its signal to parathyroid hormone (PTH)^30^^,^^31^ by preventing the store of cyclic adenosine monophosphate to build up, which is naturally associated with PTH stimulation and the expression of PTH on the cell surface membrane.^32^ Second, a key metabolic pathway for IC stimulation is the activation of intracellular calcium stores.^23^ These stores then increase activated calmodulin levels, which enhance osteoblast cell proliferation. This is the key difference to CC, where the activation of intracellular calcium is from an extracellular pathway.^23^ Thirteen studies^35^^-^47 reported that IC stimulates healing by upregulation of growth factor production including BMP-2,4,6,7, TGF-β1, and insulin growth factor-2 by osteoblasts. Figure 3c shows a summary of IC-proposed mechanism of action.

### LOE and efficacy of ES to enhance bone healing

Direct current has been utilized to aid bone healing in spinal fusion, nonunions, delayed unions, and as an adjunct for promotion of bone healing in ankle surgery (Table 3)^48^^-^81. Four studies supplied LOE-1 for utilizing DC in the treatment of spinal fusion. Direct current was found to be highly effective in the enhancement of failed spinal fusion and as an adjunct to spinal instrumentation.^51^ However, one study found no difference in fusion success after DC^49^ and another LOE-1 study showed no increase in lumbar fusion rates in patients older than 60 years after DC.^48^ Further LOE-2^52^ proved DC to be effectively employed in lumbar interbody fusion. Direct current has been effectively used as an adjunct in hindfoot fusion^62^ and revision ankle arthrosis nevertheless providing only a LOE-4^67^. The use of DC for nonunion and delayed union is limited again by just LOE-4. LOE-2 supported the use of DC in osteonecrosis of the femoral head.^12^^,^^53^

Capacitive coupling has been used to enhance bone healing in nonunions, delayed unions, and spinal fusion (Table 4)^82^^-^92. Two LOE-1 studies utilized CC for the treatment of nonunions. The first study^84^ showed CC to be highly effective for treating long bone nonunion, but the second study used it for tibial stress fractures,^82^ finding no improvement in healing time. These findings were enhanced by an LOE-4 study where athletes with lower limb stress fractures were successfully treated with CC.^86^ Furthermore, LOE-4 showed that CC was effective in healing nonunions,^87^^,^^89^^,^^90^ whereas LOE-1 has shown CC to enhance lumbar spinal fusion.^83^

Inductive coupling is extensively utilized for bone healing with 18 LOE-1 studies (Table 5)^93^^-^146. Three LOE-1 studies utilized IC for tibial nonunions. The earliest study^93^ showed no statistical difference in healing after stimulation, while later studies supported IC.^8^^,^^108^ Furthermore, LOE-4 demonstrated IC to be effective in enhancement of long bone nonunions.^123^^,^^114^^,^^110^ LOE-1 studies showed IC to be effective in enhancing healing of femoral^9^ and tibial osteotomies.^104^^,^^109^ LOE-1 proved IC ineffective for disuse of osteoporosis and bone formation during limb lengthening.^97^ Inductive coupling has been shown to aid healing of fresh fractures by LOE-1.^11^^,^^94^ Inductive coupling was supported by LOE-1 to be effective for patients undergoing interbody fusion,^106^ enhancing posterolateral lumbar fusion^105^ and increasing fusion rates in anterior cervical disectomy.^98^ LOE-1 and LOE-4 verified that IC is successful in congenital pseudoarthrosis.^107^ In contrast, LOE-1 proved IC ineffective for Perthes disease.^99^ Inductive coupling has shown to effectively enhance fusion success of hindfoot arthrodesis with one LOE-1 study^96^; nonetheless, there is conflicting inconsistent LOE-4 supporting IC for aiding fusion after ankle arthrodesis.^137^^,^^139^^,^^140^ The results of this study were used to assign grades of recommendations (Table 2). There was, however, wide study heterogeneity (Table 6).

## DISCUSSION

### Mechanisms of action of ES

The exact mechanism by which ES enhances bone repair remains underexplored. Direct current was shown to work by an electrochemical reaction at the cathode.^13^^-^22 For CC, molecular pathways and growth factors have been shown to enhance proliferation and differentiation of the osteoblast.^23^^-^26 Inductive coupling was shown to enhance osteoblast differentiation and proliferation by mechanisms involving alteration of growth factors^27^, gene expression,^28^ and transmembrane signaling.^29^ Calcium is upregulated by IC and CC, which is important in bone healing, as it has a role in the mineralization of bone and conducts the communication between cell surface receptors, antibodies, and hormones for DNA synthesis needed for bone healing.^33^^,^^34^ The upregulation of growth factor synthesis by all modes of ES acts similarly to enhance bone healing. They work in an autocrine and paracrine action^46^ to increase the cellular matrix synthesis and gene expression, which in turn increases bone cellular proliferation and differentiation, leading to enhanced callus formation and maturation.^36^^,^^37^ An overview of the mechanisms for ES is shown in Figure 3. With better understanding of the effect of ES at a molecular level, the effectiveness of ES for enhancement of bone healing in the clinical setting will be improved.

### Direct current

Using DC for spinal fusion has shown to be inconsistent with 2 LOE-1 studies^51^^,^^50^ supporting its efficacy particularly in high risk patients (smokers, those with multiple back surgeries, and multilevel fusions) and 2 LOE-1 studies showing no difference in the older patient population leaving DC only level B recommendation.^48^^,^^49^ However, one meta-analysis supports continuous 24-hour delivery of 5 to 10 µA using 2 to 4 cathodes to be effective for spinal fusion.^147^ Therefore, more studies should be carried out to support DC for spinal fusion. Moreover, DC is effective as an adjunct to foot and ankle surgery with only a level C recommendation. Because of LOE-4 being solely reported, more evidence is required because of a wide range in follow-up (9-20 weeks), small patient population, and large differences in number of surgical inventions before DC was used (range, 1-5). No studies for DC fulfill the criteria for randomized prospective double-blind clinical trial because it would involve implantation of a placebo stimulator, which is against the regulation of human research; therefore, its effect on bone healing remains questionable leaving DC only as a recommendation C for nonunion. LOE-4 supports using DC for the application of enhancing nonunions, and bone healing rates were not affected by the presence of previous osteomyelitis or the presence of previously inserted metallic fixation devices.^54^ Furthermore, rate of unions were not significantly different compared to rates after bone graft surgery.^54^ A LOE-4 study showed 10 years after DC stimulation that all fractures had remained united with normal bone remodeling, illustrating that DC is safe and effective in the long term.^59^ However, despite its effectiveness and availability, DC has fallen out of favor compared to IC and CC. Furthermore, IC and CC are noninvasive techniques affected by patient compliance unlike DC.

### Capacitive coupling

Using CC for bone healing is limited with only 2 LOE-1 studies. These studies are unreliable, as the success of CC for healing long bone nonunions by Scott and King^84^ consisted of a small sample size and had a large variety in fracture sites between control and stimulated groups. Despite Beck et al^82^ reporting good use of randomization, and blinding the outcome assessors, 86% of the patients being followed up showed no difference in the time for healing between the control and CC group. Encouragingly, LOE-4 has demonstrated CC to be effective in treating nonunions,^85^^,^^87^^,^^90^ though this unreliable evidence suggests that this application warrants further investigation leaving CC as level of recommendation as C. Using CC for spinal fusion is relatively new, with limited evidence supporting its effectiveness^83^; therefore, further studies are required though as to date giving a level of recommendation as A.

### Inductive coupling

The use of ICs for treating nonunion and delayed union has been successful.^8^^,^^108^ However, in the study by Sharrard,^8^ the age of the active group was 34.7 and in the control group, it was 45.4. Furthermore, when the results of the study of Simonis et al^108^ were adjusted for smoking, no enhancing effects were seen for IC. These limitations suggest that further clinical evidence is needed to support this application, as shown by the level of recommendation being C. Inductive coupling has been shown to be beneficial for osteotomies, but the endpoint assessment was shown to vary in 3 randomized controlled trials (RCTs) making true comparisons difficult giving an overall recommendation of B.^9^^,^^104^^,^^109^ Inductive coupling is effective in fresh fractures although supported by only 2 RCTs.^11^^,^^94^ In one such study, scintimetric analysis was a primary outcome measure.^11^ This did not reliably examine the effect of ES on patient's clinical outcomes and also failed to show any benefit in redisplacement rates between the groups. The subsequent study was limited, as the findings were based on a subgroup of patients using the device for more than 6 hours daily; therefore, the compliance of the patients was influential.^94^ Hence, overall, the recommendation remains as level B. Inductive coupling had no effect on regenerate bone during limb lengthening,^94^ even though bone loss in the segments of bone distal to the lengthening sites was significantly more marked using inactive coils, illustrating that IC can prevent bone loss adjacent to the distraction gap. However, multiple limbs were analyzed for the same patient, and the small population decreased the reliability of the results. Two RCTs agreed in supporting IC for enhancing spinal fusion, showing a recommendation of level A.^106^^,^^107^ However, in one study, the radiographic criteria for fusion required only 50% incorporation of the graft^106^ and follow-up in both studies was less than a year making definite judgment difficult. Only one LOE-1 study verified IC for congenital pseudoarthrosis,^107^ which was limited by not blinding the assessor or patients, introducing detection and performance bias, and hence better designed studies are needed giving overall assessment of recommendation of C.

Bone grafting procedures for nonunions is shown to be less successful with multiple graft procedures.^145^ However, bone grafting with ES has shown to yield good results only failing to enhance bone healing in 10% to 15% of cases.^145^ Bone grafting with DC^79^ and IC^145^ has also been shown to be effective in enhancing bone healing. Nonetheless, only 2 studies were found to use this technique.

There were certain limitations found in the studies during evaluation including the following:

1. Randomization of the RCTs was generally maintained but the allocation methods was not well-defined for the RCTs.^8^^,^^9^^,^^11^^,^^93^^,^^97^^,^^107^ The dropout rates were adequate being less than 26% on average with follow-up of the patient in the RCTs being nearly all greater than 86%,^9^^,^^11^^,^^84^^,^^93^^,^^97^^,^^108^ although few studies described the statistical power of their studies.^8^^,^^84^^,^^93^ Therefore, more prospective, appropriately powered, well-designed, randomized clinical control trials are needed demonstrating the efficacy of ES in enhancing bone healing in nonunions, delayed unions, fresh fractures, osteotomies, and spinal fusion.
2. Outcome measures for the studies varied, including clinical or radiological union or combinations of both, bone density, scintimetric values, healing success rates, and time to weight bear or consolidation (Table 6). Very few studies looked at patient outcomes, including pain, need for revision surgery, and improvement in functional status. The few studies that addressed the effect of ES on patient outcome showed no benefit in terms of pain.^8^^,^^56^^,^^61^
3. Furthermore, there is lack of agreement of a definition for a nonunion varying from 3 months to 9 months and the entry criteria amongst studies (see Tables 3–5).^84^^,^^85^^,^^88^^,^^91^ Some studies define nonunion clinically, whereas some additionally incorporate radiological criteria.^84^^,^^85^^,^^91^ Therefore, a consensus is needed for the definition of nonunion, and thus ES studies can be reliably compared.
4. The length of assessment to assess the effectiveness of ES also varied from 2 to 18 months (Table 6). The treatment time also varied as shown in Table 6. Capacitive coupling treatment ranged from 10 weeks to 6 months for between 10 and 24 hours a day and IC ranged from 3 to 18 hours daily over a period of 3 weeks to 9 months with DC being more uniform at 12 weeks.
5. There was a degree of variety in frequency and amplitude within the type of ES subgroups (Tables 3–5). Most IC devices reported a similar frequency between 15 and 75 kHz.^9^^,^^10^^,^^11^^,^^93^^,^^94^^,^^110^^,^^114^^,^^115^^,^^119^ Direct current was generally reported using 20 µA across 4 cathodes^53^^,^^54^^,^^55^^,^^56^ though there was heterogeneity, as 2 studies reported 40 µA^48^^,^^65^ and 3 studies used 10 µA.^51^^,^^57^^,^^75^ Capacitive coupling in approximately half of the studies reported at 60 kHz at 5 V.^82^^,^^87^^,^^88^^,^^90^^,^^91^
6. Few trials had more than 80 patients; for example, there were only 10 patients in DC and IC and 1 patient in CC, which was for spinal fusion (see patient number in Tables 3–5).
7. Only 3 studies compared the clinical efficacy of different methods of ES,^49^^,^^63^^,^^86^ which may be due to small number of patients used in ES studies. These studies are required, as they offer the potential to illustrate the most beneficial mode of ES for each orthopedic clinical problem.

In conclusion, the exact mechanism by which ES enhances bone repair is still not fully understood and needs more investigation. However, to date, DC has been documented to work by an electrochemical reaction at the cathode, and CC and IC have shown to work by alteration of growth factors and transmembrane signaling. In an era of evidence-based medicine, fracture-healing management should be based on the best available evidence to ensure high-quality, safe, and cost-effective treatment. Therefore, considering the widespread usage of ES to aid bone healing in clinical practice, our analysis shows that there have been few good quality clinical studies to support their use. This is demonstrated by the low recommendation assigned to ES for different orthopedic conditions requiring bone healing except spinal fusion (Table 2). Therefore, the optimal regime for ES treatment for bone healing needs to be further defined and standardized. Moreover, clinical studies need to have uniform outcomes and defined criteria on the effect of ES on clinical outcomes including improvement in pain, activities of daily living, and need for revision surgery. Overall, the evidence to date implies that further studies are needed to support and optimize the clinical application of ES for bone healing.

## Figures and Tables

**Figure 1:**
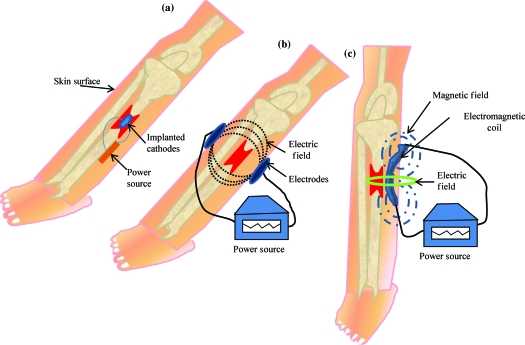
The three methods of administering electric stimulation are shown in this diagram. (*a*) Direct current (DC): A cathode is implanted at the fracture site which is attached to either a subcutaneous power source or an external power source to generate an electric field at the fracture site. (*b*) Capacitive coupling(CC): Two capacitive coupled electrodes are situated on the skin on either sides of the fracture site. An external power source is then attached to the electrodes, which induces an electric field at the fracture site. (*c*) Inductive coupling (IC): An electromagnetic current carrying coil is placed on the skin overlying the fracture site, which is attached to an external power source. The coil generates a magnetic field, which induces an electrical field at the fracture site.

**Table 1:** A table illustrating some of the clinical electrical stimulation devices used today which have been FDA approved.*

Company	Device Name	Electrical Type	Description of Product
Orthofix	Physio-Stim Lite	PEMF	Noninvasive device for nonunions for both short and long bones
Orthofix	Cervical and Spinal-Stim Lite	PEMF	Noninvasive device for spinal fusion
Biomet	EBI bone healing system	PEMF	Noninvasive device for nonunion fractures, failed fusions and congenital pseudarthrosis
Biomet	OrthoPak 2 bone growth stimulator	CC	Noninvasive device for nonunion fractures
Biomet	SpinalPak bone growth stimulator	CC	Noninvasive device for spinal fusion for one to two levels
Biomet	OsteoGen and OsteoGen-D	DC	Surgically implanted device for nonunions and may also be used as an adjunct to internal/external fixation and autograft
Biomet	SpF implantable spine fusion stimulator	DC	The SpF-2T and SpF-4T are indicated for spinal fusion of one or two levels, while the SpF-XL and SpF-XL IIb are indicated for fusion of three or more levels

*DC indicates direct current; CC, capacitive coupling; FDA, Food and Drug Administration; PEMF, pulsed electromagnetic field.

**Table 2:** Grade of recommendation for each mode of electrical stimulation for each type of bone healing diagnosis based on Oxford Centre Level of Evidence Recommendation.

	Level of Recommendation
Direct current	
Spinal fusion	B
Ankle/foot union	C
Osteonecrosis of the femoral head	B
Nonunion	C
Capacitive coupling	
Spinal fusion	A
Nonunion	C
Pulsed electromagnetic field	
Spinal fusion	A
Ankle/foot union	C
Osteonecrosis of the femoral head	C
Nonunion	C
Osteotomy	B
Fresh fracture	B
Congenital pseudoarthrosis	C

DC indicates direct current; CC, capacitive coupling; PEMF, pulsed electromagnetic field.

**Table 3:** Clinical studies reviewed for DC.*

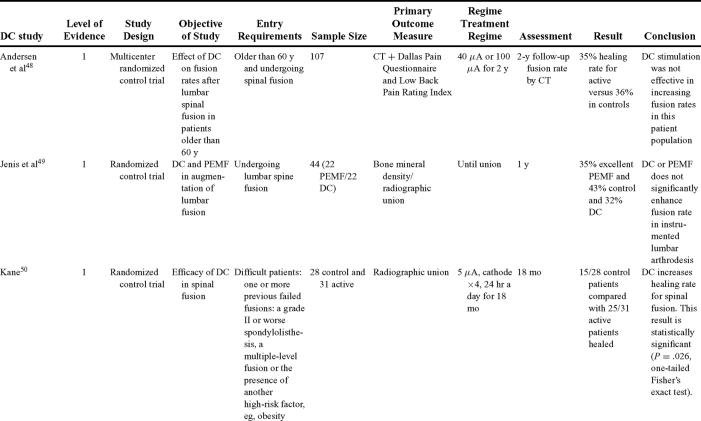
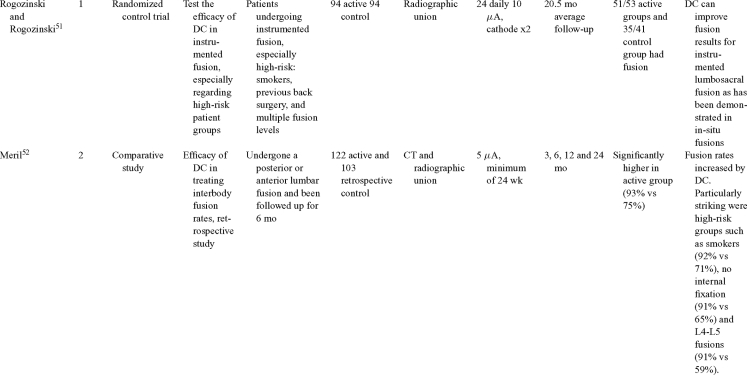
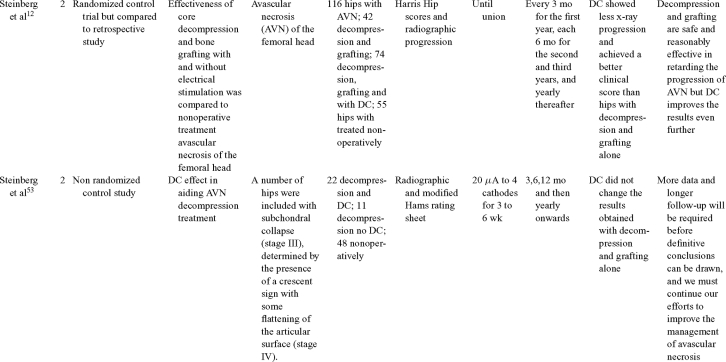
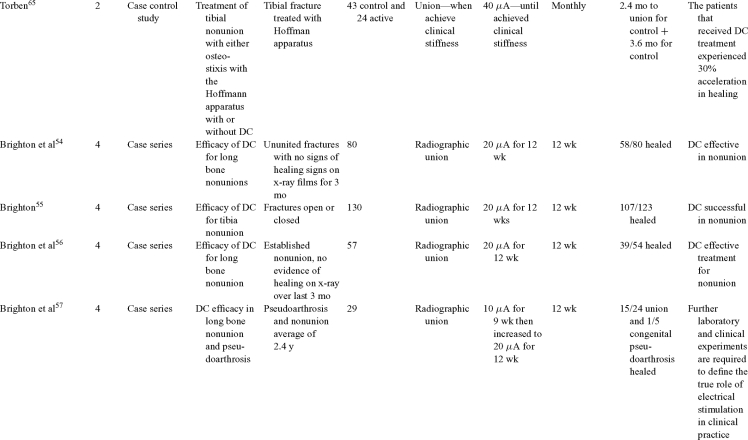
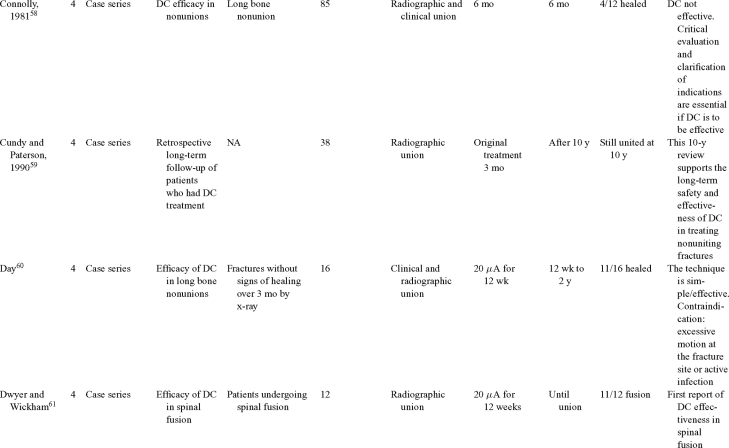
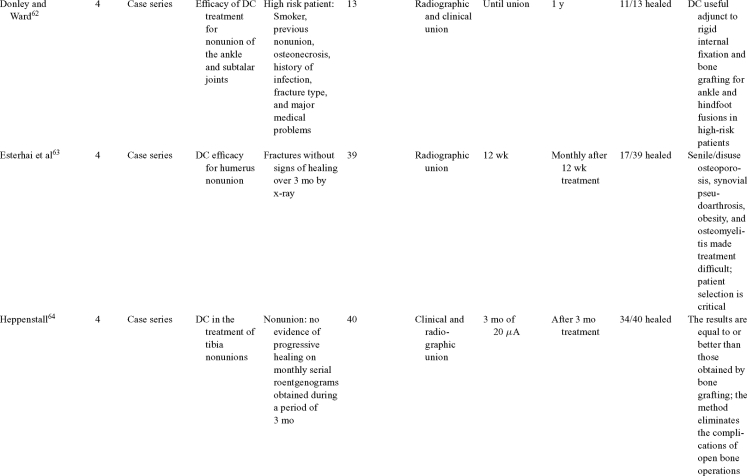
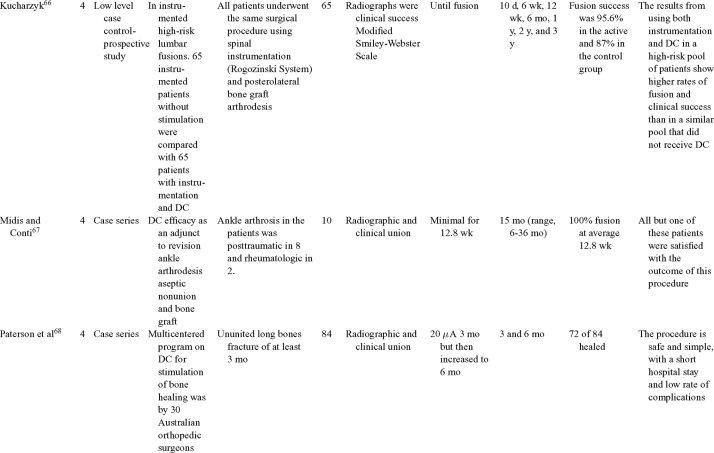
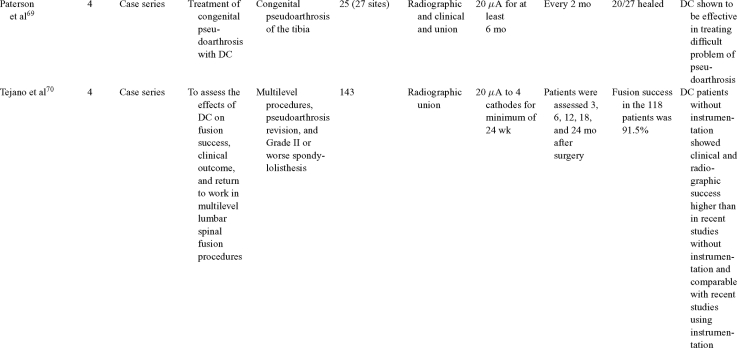
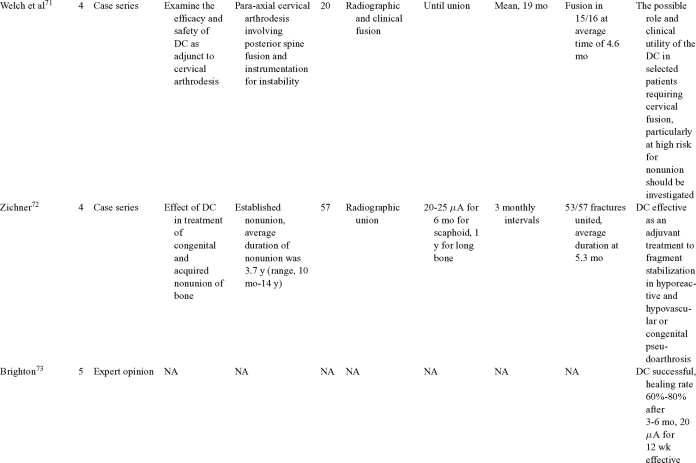
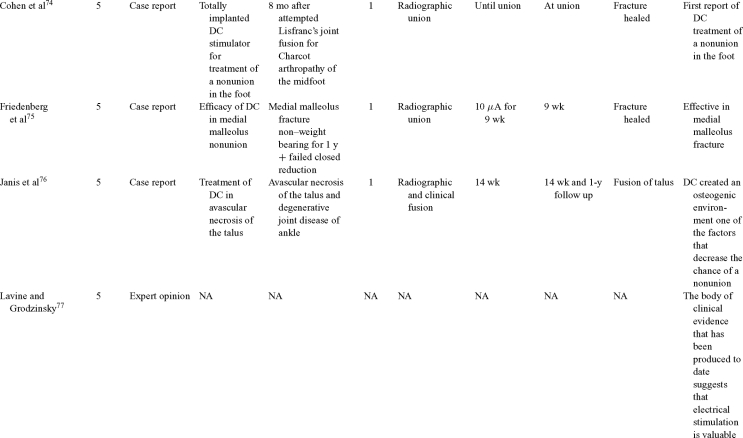
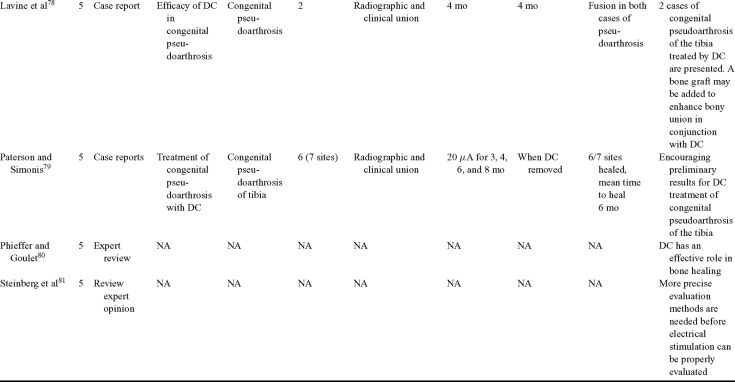

*DC indicates direct current; CC, capacitive coupling; FDA, Food and Drug Administration; NA, not applicable; PEMF, pulsed electromagnetic field.

**Table 4:** Clinical studies reviewed for capacitive coupling (CC).*

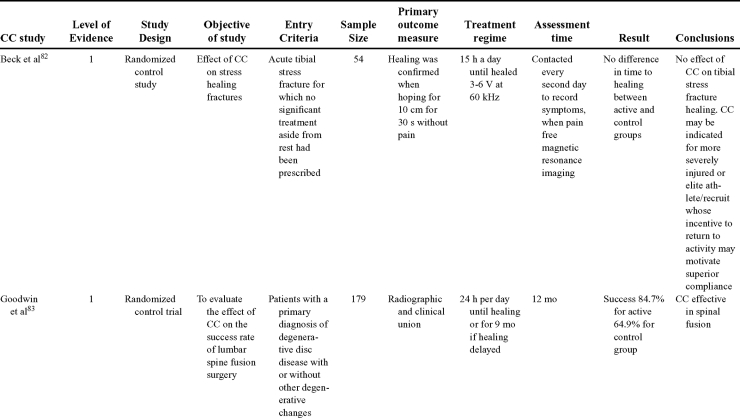
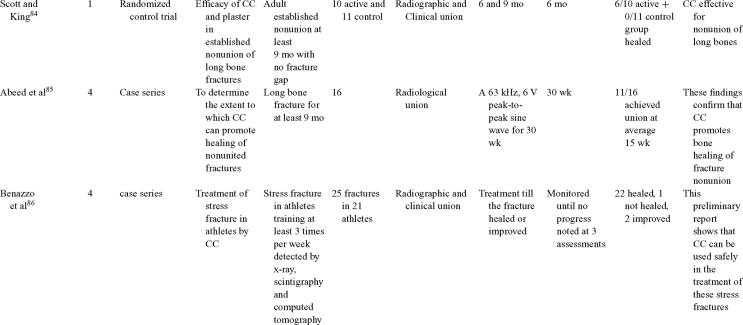
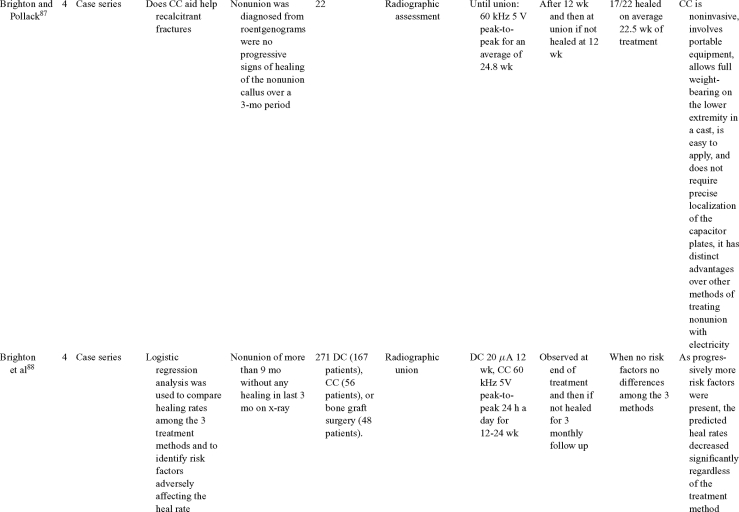
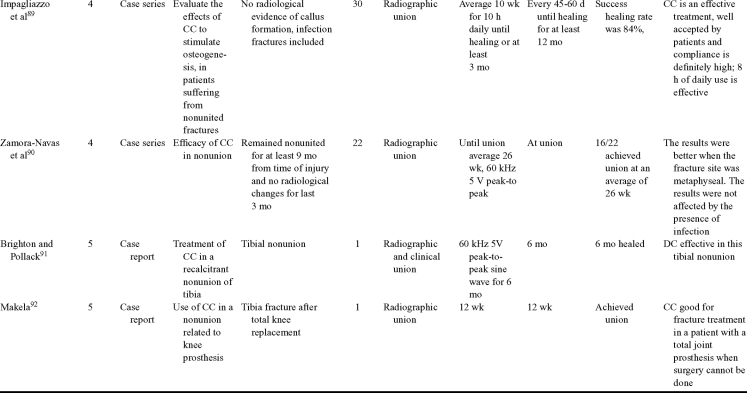

**Table 5:** Clinical studies reviewed for Inductive coupling (IC) which is also referred to as pulsed electromagnetic field.*

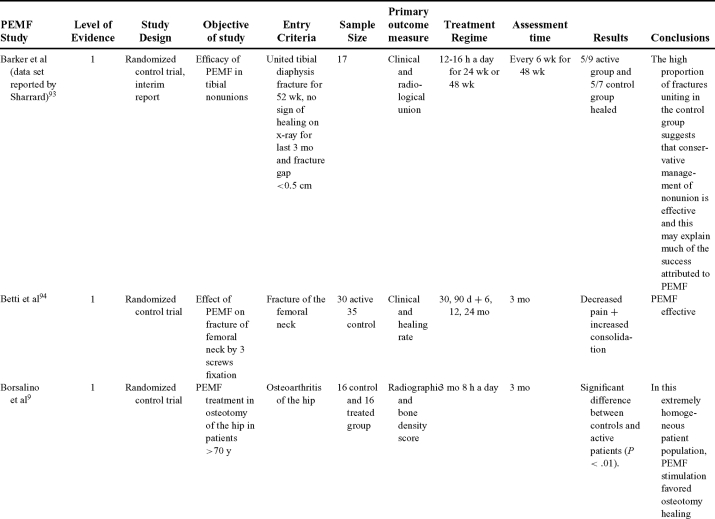
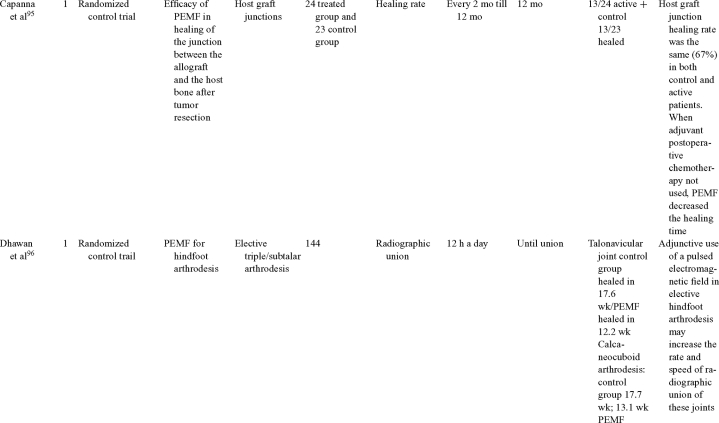
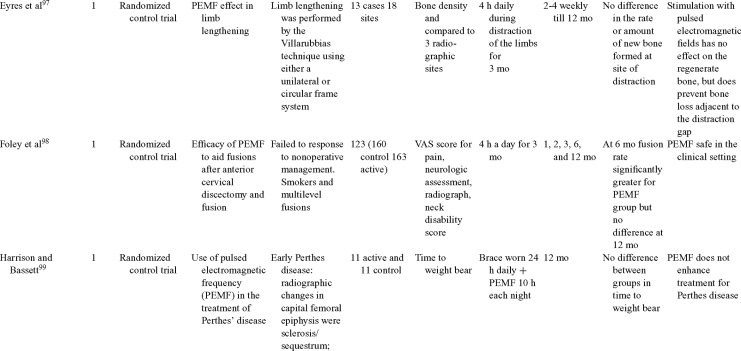
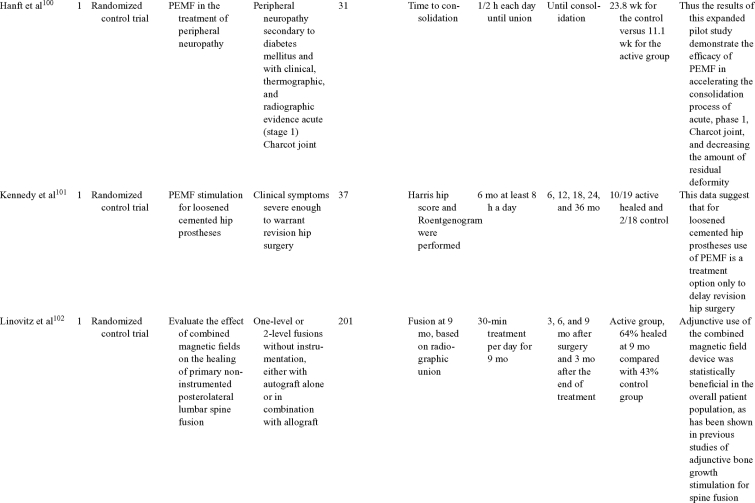
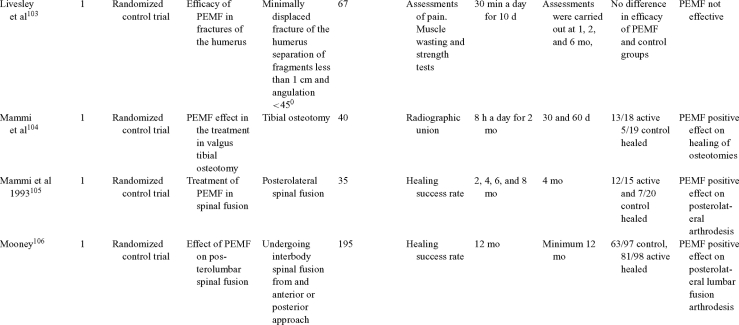
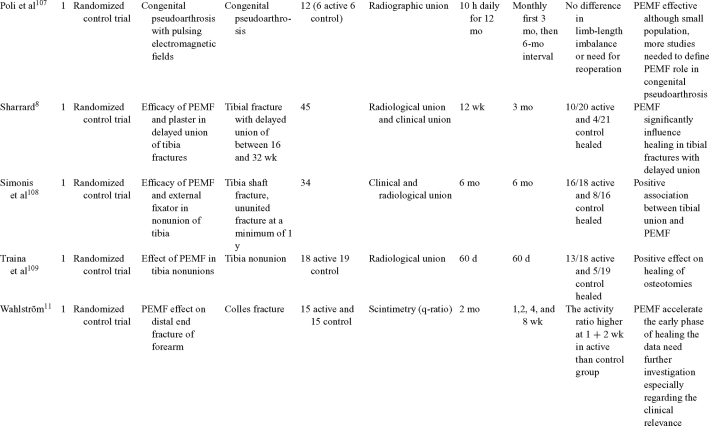
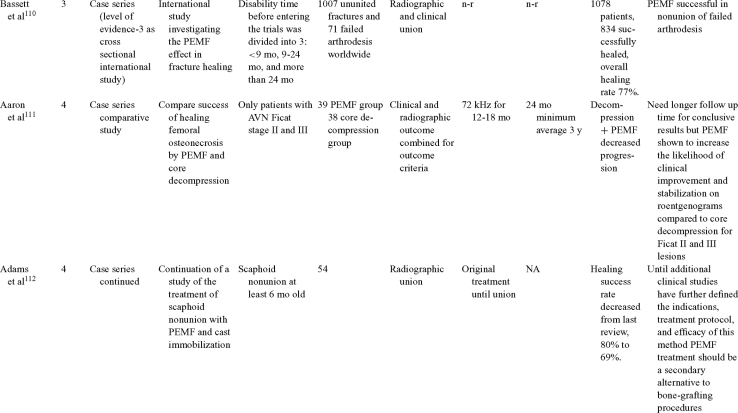
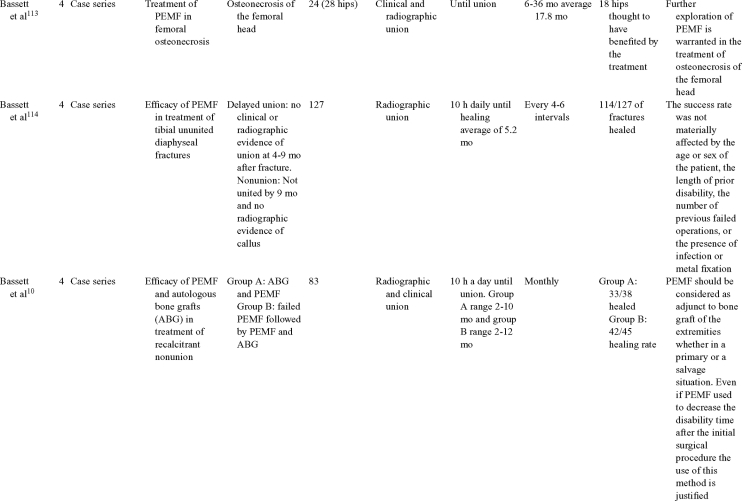
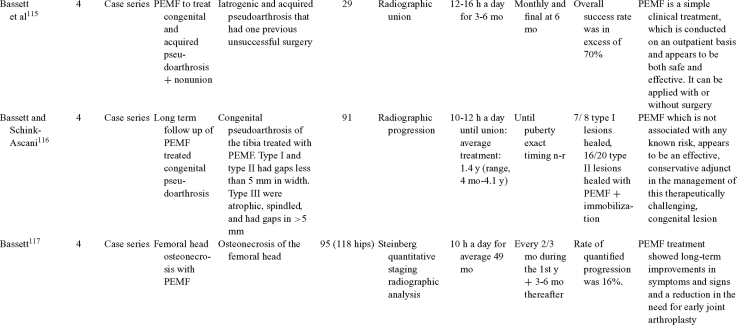
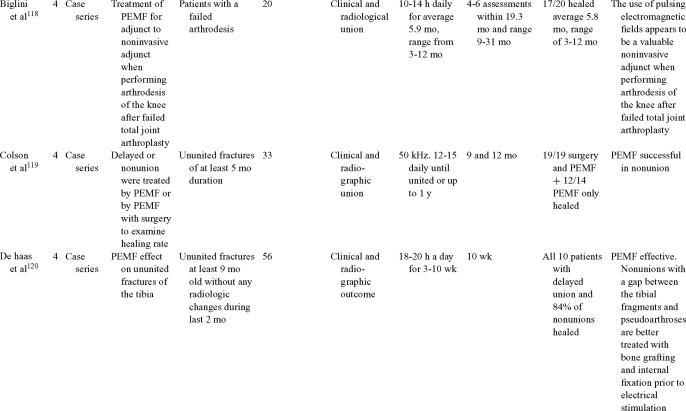
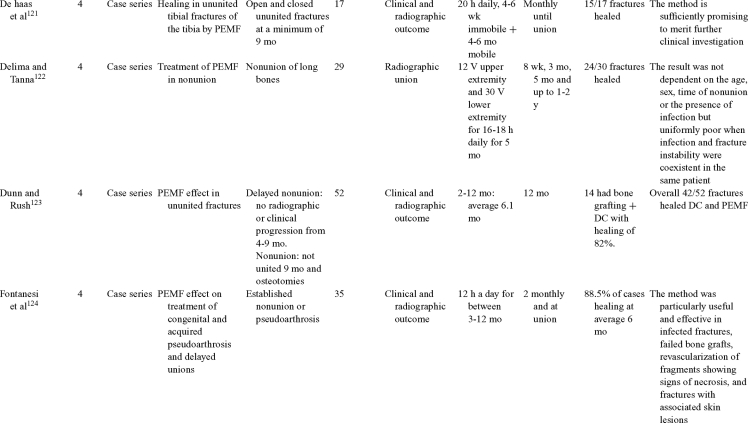
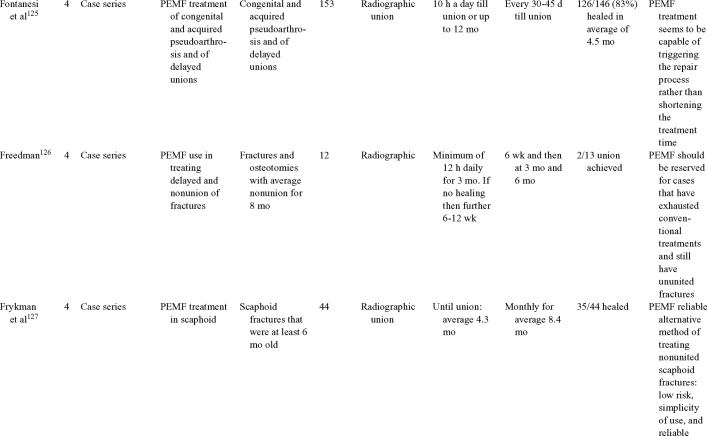
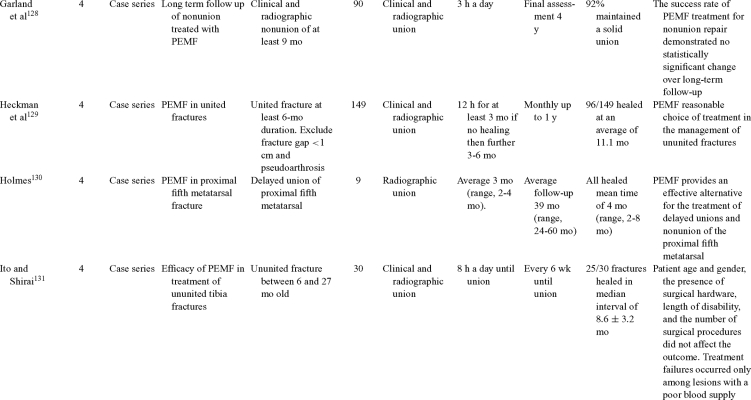
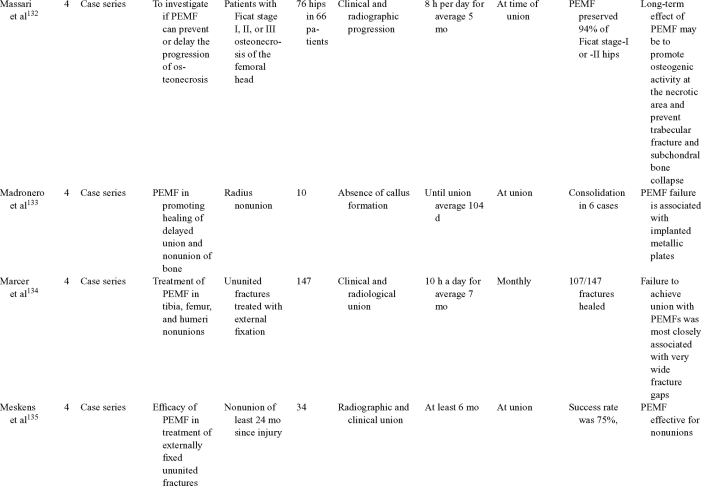
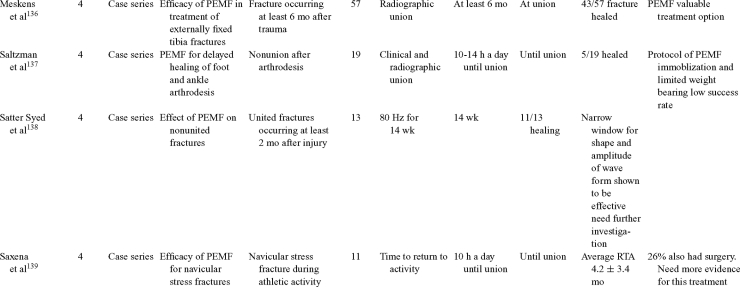
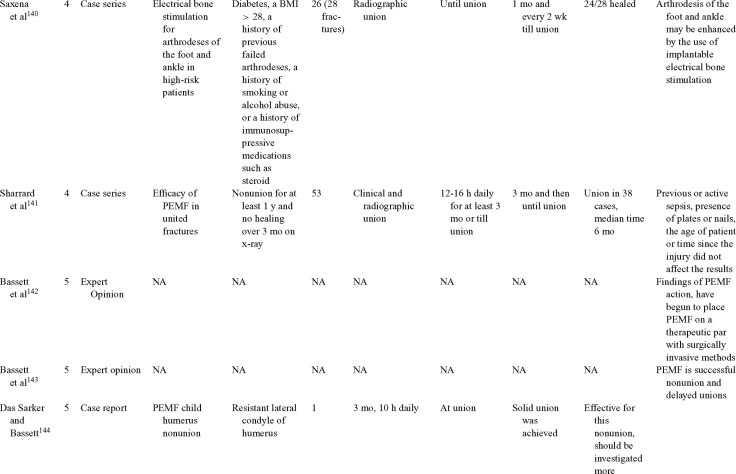
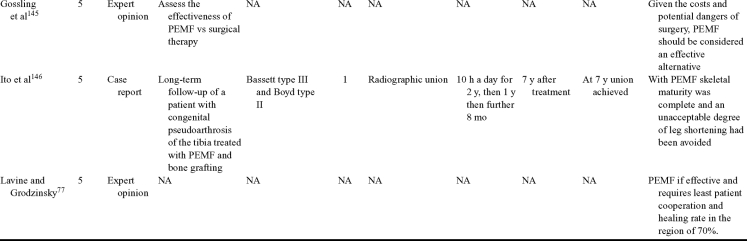

**Table 6:** Analysis of heterogeneity of clinical studies, including evaluation of treatment time, outcome measure, and assessment time follow-up for all 3 types of electrical stimulation.*

	Number of studies for DC (n)		Number of studies for CC (n)	Number of studies for PEMF (n)
		**A**		
Treatment time				
≥3 mo duration	11		1	15
3- to ≥6-mo duration	4		4	9
6-mo to ≥ 1-y duration	5		1	10
>1 y duration	3		0	2
Until union duration	7		5	14
Not state duration of treatment	1		0	5
24 h per day	NA		2	1
10-16 h per day	NA		2	25
0.5-8 h	NA		0	10
Not state time per day	NA		7	19
		**B**		
Outcome measure				
Radiographic	19		5	22
Clinical	1		0	7
Radiographic and Clinical	11		5	21
Hoping for 30 s	0		1	0
Bone density	0		0	2
Time to weight bear	0		0	1
Scintimetric analysis	0		0	1
Time to consolidation	0		0	1
		**C**		
Assessment time follow-up				
≥3 mo	8		2	7
3 to ≥6 mo	3		3	5
6 mo to ≥1 y	7		2	11
>1 y	7		0	6
Until union	6		4	25
Not state	00 0		0	1

*DC indicates direct current; CC, capacitive coupling; PEMF, pulsed electromagnetic field.

## References

[1] Ryaby JT (1998). Clinical effects of electromagnetic and electric fields on fracture healing. Clin Orthop.

[2] Fukada E, Yasuda I (1957). On the piezoelectric effect of bone. J Phys Soc Japan.

[3] Black J (1987). Electrical Stimulation: Its Role in Growth, Repair, and Remodeling of the Musculoskeletal System.

[4] Evans RD, Foltz D, Foltz K (2001). Electrical stimulation with bone and wound healing. Clin Podiatr Med Surg.

[5] Aaron RK, Ciombor DM, Simon BJ (2004). Treatment of Nonunions with electric and electromagnetic fields. Clin Orthop Relat Res.

[6] Aaron RK, Steinberg ME (1991). Electrical stimulation of osteonecrosis of the femoral head. Semin Arthroplasty.

[7] Yasuda I (1953). Fundamental aspects of fracture treatment. J Kyoto Med Soc.

[8] Sharrard WJ (1990). A double-blind trial of pulsed electromagnetic fields for delayed union of tibial fractures. J Bone Joint Surg Br.

[9] Borsalino G, Bagnacani M, Bettati E (1988). Electrical stimulation of human femoral intertrochanteric osteotomies: double-blind study. Clin Orthop Relat Res.

[10] Bassett CA, Mitchell SN, Schink MM (1982). Treatment of therapeutically resistant nonunions with bone grafts and pulsing electromagnetic fields. J Bone Joint Surg Am.

[11] Wahlström O (1984). Stimulation of fracture healing with electromagnetic fields of extremely low frequency (EMF of ELF). Clin Orthop Relat Res.

[12] Steinberg ME, Brighton CT, Corces A (1989). Osteonecrosis of the femoral head: results of core decompression and grafting with and without electrical stimulation. Clin Orthop Relat Res.

[13] Baranowski TJ, Black J, Blank M, Findl E (1987). The mechanism of faradic stimulation of osteogenesis. Mechanistic Approaches to Interactions of Electric and Electromagnetic Fields With Living Systems.

[14] Bassett CA, Herrmann I (1961). Influence of oxygen concentration and mechanical factors on differentiation of connective tissues in vitro. Nature.

[15] Bodamyali T, Kanczler JM, Simon B (1999). Effect of faradic products on direct current-stimulated calvarial organ culture calcium levels. Biochem Biophys Res Commun.

[16] Brighton CT, Adler S, Black J (1975). Cathodic oxygen consumption and electrically induced osteogenesis. Clin Orthop Relat Res.

[17] Brighton CT, Ray RD, Soble LW (1965). In vitro epiphyseal-plate growth in various oxygen tensions. J Bone Joint Surg Am.

[18] Cho M, Hunt TK, Hussain MZ (2001). Hydrogen peroxide stimulates macrophage vascular endothelial growth factor release. Am J Physiol Heart Circ Physiol.

[19] Gan J, Fredericks D, Glazer P (2004). Direct current and capacitive coupling electrical stimulation upregulates osteopromotive factors for spinal fusions. Orthop J Harvard Med School.

[20] Steinbeck MJ, Kim JK, Trudeau MJ (1998). Involvement of hydrogen peroxide in the differentiation of clonal HD-11EM cells into osteoclast-like cells. J Cell Physiol.

[21] Wang Q, Shizen Z, Xie Y (1995). Electrochemical reactions during constant DC current stimulation: an in vitro experiment with cultured rat calvarial cell. Electro Magnetobiol.

[22] Bushinsky DA (1996). Metabolic alkalosis decreases bone calcium efflux by suppressing osteoclasts and stimulating osteoblasts. Am J Physiol Renal Physiol.

[23] Brighton CT, Wang W, Seldes R (2001). Signal transduction in electrically stimulated bone cells. J Bone Joint Surg Am.

[24] Lorich DG, Brighton CT, Gupta R (1998). Biochemical pathway mediating the response of bone cells to capacitive coupling. Clin Orthop Relat Res.

[25] Wang Z, Clark CC, Brighton CT (2006). Up-regulation of bone morphogenetic proteins in cultured murine bone cells with use of specific electric fields. J Bone Joint Surg Am.

[26] Zhuang H, Wang W, Seldes RM (1997). Electrical stimulation induces the level of TGF-beta1 mRNA in osteoblastic cells by a mechanism involving calcium/calmodulin pathway. Biochem Biophys Res Commun.

[27] Yen-Patton GP, Patton WF, Beer DM (1988). Endothelial cell response to pulsed electromagnetic fields: stimulation of growth rate and angiogenesis in vitro. J Cell Physiol.

[28] De Mattei M, Gagliano N, Moscheni C (2005). Changes in polyamines, c-myc and c-fos gene expression in osteoblast like-cells exposed to pulsed electromagnetic fields. Bioelectromagnetics.

[29] Chiabrera A, Grattarola M, Viviani R (1984). Interaction between electromagnetic fields and cells: microelectrophoretic effect on ligands and surface receptors. Bioelectromagnetics.

[30] Luben RA, Cain C, Chen M (1982). Effects of electromagnetic stimulation on bone and bone cells in vitro: inhibition to parathyroid hormone by low energy low frequency fields. Pro Natl Sci U S A.

[31] Spadaro JA, Bergstrom WH (2002). In vivo and in vitro effects of a pulsed electromagnetic field on net calcium flux in rat calvarial bone. Calcif Tissue Int.

[32] Cain CD, Adey WR, Luben RA (1987). Evidence that pulsed electromagnetic fields inhibit coupling of adenylate cyclase by parathyroid hormone in bone cells. J Bone Miner Res.

[33] Adey WR (1993). Biological effects of electromagnetic fields. J Cell Biochem.

[34] Luben RA (1991). Effects of low-energy electromagnetic fields (pulsed and DC) on membrane signal transduction processes in biological systems. Health Phys.

[35] Aaron RK, Ciombor DM, Keeping H (1999). Power frequency fields promote cell differentiation coincident with an increase in transforming growth factor-beta(1) expression. Bioelectromagnetics.

[36] Aaron RK, Wang S, Ciombor DM (2002). Upregulation of basal TGFbeta1 levels by EMF coincident with chondrogenesis: implications for skeletal repair and tissue engineering. J Orthop Res.

[37] Bodamyali T, Bhatt B, Hughes FJ (1998). Pulsed electromagnetic fields simultaneously induce osteogenesis and upregulate transcription of bone morphogenetic proteins 2 and 4 in rat osteoblasts in vitro. Biochem Biophys Res Commun.

[38] Fitzsimmons RJ, Ryaby JT, Mohan S (1995). Combined Magnetic fields increase insulin-like growth-II in TE85 human osteosarcoma bone cell cultures. Endocrinology.

[39] Guerkov HH, Lohmann CH, Liu Y (2001). Pulsed electromagnetic fields increase growth factor release by nonunion cells. Clin Orthop Relat Res.

[40] Lohmann CH, Schwartz Z, Liu Y (2000). Pulsed electromagnetic field stimulation of MG63 osteoblast-like cells affects differentiation and local factor production. J Orthop Res.

[41] Lohmann CH, Schwartz Z, Liu Y (2003). Pulsed electromagnetic fields affect phenotype and connexin 43 protein expression in MLO-Y4 osteocyte-like cells and ROS 17/2.8 osteoblast-like cells. J Orthop Res.

[42] Nagai M, Ota M (1994). Pulsating electromagnetic field stimulates mRNA expression of bone morphogenetic protein-2 and -4. J Dent Res.

[43] Ryaby J, Fitzsimmons R, Kin N (1994). The role of insulin-like growth factors II in magnetic field regulation of bone formation: the role of insulin-like growth factors II in magnetic field regulation of bone formation. Biolectrochem Bioeng.

[44] Tepper OM, Callaghan MJ, Chang EI (2004). Electromagnetic fields increase in vitro and in vivo angiogenesis through endothelial release of FGF-2. FASEB J.

[45] Yajima A, Ochi M, Hirose Y (1994). Effects of pulsing electromagnetic fields on gene expression of bone morphogenetic protein-2 and -4. J Dent Res.

[46] Aaron RK, Boyan BD, Ciombor DM (2004). Stimulation of growth factor synthesis by electric and electromagnetic fields. Clin Orthop Relat Res.

[47] Schwartz Z, Simon BJ, Duran MA (2008). Pulsed electromagnetic fields enhance BMP-2 dependent osteoblastic differentiation of human mesenchymal stem cells. J Orthop Res.

[48] Andersen T, Christensen FB, Egund N (2009). The effect of electrical stimulation on lumbar fusion in older patients: a randomised controlled multicenter trial, part 2: fusion rates. Spine.

[49] Jenis LG, An HS, Stein R (2000). Prospective comparison of the effect of direct current electrical stimulation and pulsed electromagnetic fields on instrumented posterolateral lumbar arthrodesis. J Spinal Disord.

[50] Kane WJ (1988). Direct current electrical bone growth stimulation for spinal fusion. Spine.

[51] Rogozinski A, Rogozinski C (1996). Efficacy of implanted bone growth stimulation in instrumented lumbosacral spinal fusion. Spine.

[52] Meril AJ (1994). Direct current stimulation of allograft in anterior and posterior lumbar interbody fusions. Spine.

[53] Steinberg ME, Brighton CT, Hayken GD (1989). Early results in the treatment of avascular necrosis of the femoral head with electrical stimulation. Orthop Clin North Am.

[54] Brighton CT, Black J, Friedenberg ZB (1981). A multicenter study of the treatment of nonunion with constant direct current. J Bone Joint Surg Am.

[55] Brighton CT (1981). Treatment of nonunion of the tibia with constant direct current (1980 Fitts Lecture, A.A.S.T.). J Trauma.

[56] Brighton CT, Friedenberg ZB, Mitchell EI, Booth RE (1977). Treatment of nonunion with constant direct current. Clin Orthop Relat Res.

[57] Brighton CT, Friedenberg ZB, Zemsky LM (1975). Direct-current stimulation of nonunion and congenital pseudarthrosis: exploration of its clinical application. J Bone Joint Surg Am.

[58] Connolly JF (1981). Selection, evaluation and indications for electrical stimulation of ununited fractures. Clin Orthop Relat Res.

[59] Cundy PJ, Paterson DC (1990). A ten-year review of treatment of delayed union and nonunion with an implanted bone growth stimulator. Clin Orthop Relat Res.

[60] Day L (1981). Electrical stimulation in the treatment of ununited fractures. Clin Orthop Relat Res.

[61] Dwyer AF, Wickham GG (1974). Direct current stimulation in spinal fusion. Med J Aust.

[62] Donley BG, Ward DM (2002). Implantable electrical stimulation in high-risk hindfoot fusions. Foot Ankle Int.

[63] Esterhai JL, Brighton CT, Heppenstall RB (1986). Nonunion of the humerus. Clinical, roentgenographic, scintigraphic, and response characteristics to treatment with constant direct current stimulation of osteogenesis. Clin Orthop Relat Res.

[64] Heppenstall RB (1983). Constant direct-current treatment for established nonunion of the tibia. Clin Orthop Relat Res.

[65] Torben EJ (1977). Electrical stimulation of human fracture healing by means of a slow pulsating asymmetrical direct current. Clin Orthop Relat Res.

[66] Kucharzyk DW (1999). A controlled prospective outcome study of implantable electrical stimulation with spinal instrumentation in a high-risk spinal fusion population. Spine.

[67] Midis N, Conti S (2000). Revision ankle arthrodesis. Foot and Ankle Int.

[68] Paterson DC, Lewis GN, Cass CA (1980). Treatment of delayed union and nonunion with an implanted direct current stimulator. Clin Orthop Relat Res.

[69] Paterson DC, Lewis GN, Cass CA (1985). Treatment of congenital pseudarthrosis of the tibia with direct current stimulation. Clin Orthop Relat Res.

[70] Tejano NA, Puno R, Ignacio JM (1996). The use of implantable direct current stimulation in multilevel spinal fusion without instrumentation: a prospective clinical and radiographic evaluation with long-term follow-up. Spine.

[71] Welch WC, Willis SL, Gerszten PC (2004). Implantable direct current stimulation in para-axial cervical arthrodesis. Adv Ther.

[72] Zichner L (1981). Repair of nonunions by electrically pulsed current stimulation. Clin Orthop Relat Res.

[73] Brighton CT (1981). The treatment of nonunions with electricity. J Bone Joint Surg Am.

[74] Cohen M, Roman A, Lovins JE (1993). Totally implanted direct current stimulator as treatment for a nonunion in the foot. J Foot Ankle Surg.

[75] Friedenberg ZB, Harlow MC, Brighton CT (1971). Healing of nonunion of the medial malleolus by means of direct current: a case report. J Trauma.

[76] Janis L, Krawetz L, Wagner S (1996). Ankle and subtalar fusion utilizing a tricortical bone graft, bone stimulator, and external fixator after avascular necrosis of the talus. J Foot Ankle Surg.

[77] Lavine LS, Grodzinsky AJ (1987). Electrical stimulation of repair of bone. J Bone Joint Surg Am.

[78] Lavine LS, Lustrin I, Shamos MH (1977). Treatment of congenital pseudarthrosis of the tibia with direct current. Clin Orthop Relat Res.

[79] Paterson DC, Simonis RB (1985). Electrical stimulation in the treatment of congenital pseudarthrosis of the tibia. J Bone Joint Surg Br.

[80] Phieffer LS, Goulet JA (2006). Delayed unions of the tibia. J Bone Joint Surg Am.

[81] Steinberg ME, Brighton CT, Steinberg DR (1984). Treatment of avascular necrosis of the femoral head by a combination of bone grafting, decompression, and electrical stimulation. Clin Orthop Relat Res.

[82] Beck BR, Matheson GO, Bergman G (2008). Do capacitively coupled electric fields accelerate tibial stress fracture healing? A randomized controlled trial. Am J Sports Med.

[83] Goodwin CB, Brighton CT, Guyer RD (1999). A double-blind study of capacitively coupled electrical stimulation as an adjunct to lumbar spinal fusions. Spine.

[84] Scott G, King JB (1994). A prospective, double-blind trial of electrical capacitive coupling in the treatment of nonunion of long bones. J Bone Joint Surg Am.

[85] Abeed RI, Naseer M, Abel EW (1998). Capacitively coupled electrical stimulation treatment: results from patients with failed long bone fracture unions. J Orthop Trauma.

[86] Benazzo F, Mosconi M, Beccarisi G (1995). Use of capacitive coupled electric fields in stress fractures in athletes. Clin Orthop Relat Res.

[87] Brighton CT, Pollack SR (1985). Treatment of recalcitrant nonunion with a capacitively coupled electrical field: a preliminary report. J Bone Joint Surg Am.

[88] Brighton CT, Shaman P, Heppenstall RB (1995). Tibial nonunion treated with direct current, capacitive coupling, or bone graft. Clin Orthop Relat Res.

[89] Impagliazzo A, Mattei A, Spurio Pompili GF (2006). Treatment of nonunited fractures with capacitively coupled electric field. J Orthop Traumatology.

[90] Zamora-Navas P, Borras Verdera A, Antelo Lorenzo R (1995). Electrical stimulation of bone nonunion with the presence of a gap. Acta Orthop Belg.

[91] Brighton CT, Pollack SR (1984). Treatment of nonunion of the tibia with a capacitively coupled electrical field. J Trauma.

[92] Makela EA (1992). Capacitively coupled electrical field in the treatment of a leg fracture after total knee replacement. J Orthop Trauma.

[93] Barker AT, Dixon RA, Sharrard WJ (1984). Pulsed magnetic field therapy for tibial nonunion: interim results of a double-blind trial. Lancet.

[94] Betti E MS, Cadossi R, Faldini C, Besani F (1999). Effect of stimulation by low-frequency pulsed electromagnetic fields in subjects with fracture of the femoral neck. Electricity and Magnetism in Biology and Medicine.

[95] Capanna R, Donati D, Masetti C (1994). Effect of electromagnetic fields on patients undergoing massive bone graft following bone tumour resection: a double blind study. Clin Orthop Relat Res.

[96] Dhawan SK, Conti SF, Towers J (2004). The effect of pulsed electromagnetic fields on hindfoot arthrodesis: a prospective study. J Foot Ankle Surg.

[97] Eyres KS, Saleh M, Kanis JA (1996). Effect of pulsed electromagnetic fields on bone formation and bone loss during limb lengthening. Bone.

[98] Foley KT, Mroz TE, Arnold PM (2008). Randomized, prospective, and controlled clinical trial of pulsed electromagnetic field stimulation for cervical fusion. Spine J.

[99] Harrison MH, Bassett CA (1997). The results of a double-blind trial of pulsed electromagnetic frequency in the treatment of Perthes' disease. J Pediatr Orthop.

[100] Hanft JR, Goggin JP, Landsman A (1998). The role of combined magnetic field bone growth stimulation as an adjunct in the treatment of neuroarthropathy/Charcot joint: an expanded pilot study. J Foot Ankle Surg.

[101] Kennedy WF, Roberts CG, Zuege RC (1993). Use of pulsed electromagnetic fields in treatment of loosened cemented hip prostheses: a double-blind trial. Clin Orthop Relat Res.

[102] Linovitz RJ, Pathria M, Bernhardt M (2002). Combined magnetic fields accelerate and increase spine fusion: a double-blind, randomized, placebo controlled study. Spine.

[103] Livesley PJ, Mugglestone A, Whitton J (1992). Electrotherapy and the management of minimally displaced fracture of the neck of the humerus. Injury.

[104] Mammi GI, Rocchi R, Cadossi R (1993). The electrical stimulation of tibial osteotomies. Double-blind study. Clin Orthop Relat Res.

[105] Mammi GI, Rocchi R, DiSilvestre M, Blank M (1993). Effect of electromagnetic fields on spinal fusion: a prospective study with a control group. Electricity and Magnetism in Biology and Medicine.

[106] Mooney V (1990). A randomised double-blind prospective study of the efficacy of pulsed electromagnetic fields for interbody lumbar fusions. Spine.

[107] Poli G, Dal Monte A, Cosco F (1985). Treatment of congenital pseudarthrosis with endomedullary nail and low frequency pulsing electromagnetic fields: a controlled study. Electromagnetic Biology and Medicine.

[108] Simonis RB, Parnell EJ, Ray PS (2003). Electrical treatment of tibial nonunion: a prospective, randomised, double-blind trial. Injury.

[109] Traina G, Sollazzo V, Massari L, Bersani F (1999). Electrical stimulation of tibial osteotomies: a double blind study. Electricity and Magnetism in Biology and Medicine.

[110] Bassett CA, Mitchell SN, Gaston SR (1982). Pulsing electromagnetic field treatment in ununited fractures and failed arthrodeses. JAMA.

[111] Aaron RK, Lennox D, Bunce GE (1989). The conservative treatment of osteonecrosis of the femoral head: a comparison of core decompression and pulsing electromagnetic fields. Clin Orthop Relat Res.

[112] Adams BD, Frykman GK, Taleisnik J (1992). Treatment of scaphoid nonunion with casting and pulsed electromagnetic fields: a study continuation. J Hand Surg Am.

[113] Bassett CA, Schink MM, Mitchell SN (1983). Treatment of osteonecrosis of the hip with specific, pulsed electromagnetic fields (ICs): a preliminary clinical report. Hip.

[114] Bassett CA, Mitchell SN, Gaston SR (1981). Treatment of ununited tibial diaphyseal fractures with pulsing electromagnetic fields. J Bone Joint Surg Am.

[115] Bassett CA, Pilla AA, Pawlu RJ (1977). A non-operative salvage of surgically-resistant pseudarthroses and nonunions by pulsing electromagnetic fields: a preliminary report. Clin Orthop Relat Res.

[116] Bassett CA, Schink-Ascani M (1991). Long-term pulsed electromagnetic field (IC) results in congenital pseudarthrosis. Calcif Tissue Int.

[117] Bassett CA (1989). Fundamental and practical aspects of therapeutic uses of pulsed electromagnetic fields (ICs). Crit Rev Biomed Eng.

[118] Bigliani LU, Rosenwasser MP, Caulo N (1983). The use of pulsing electromagnetic fields to achieve arthrodesis of the knee following failed total knee arthroplasty: a preliminary report. J Bone Joint Surg Am.

[119] Colson DJ, Browett JP, Fiddian NJ (1988). Treatment of delayed- and nonunion of fractures using pulsed electromagnetic fields. J Biomed Eng.

[120] de Haas WG, Beaupre A, Cameron H (1986). The Canadian experience with pulsed magnetic fields in the treatment of ununited tibial fractures. Clin Orthop Relat Res.

[121] de Haas WG, Watson J, Morrison DM (1980). Non-invasive treatment of ununited fractures of the tibia using electrical stimulation. J Bone Joint Surg Br.

[122] Delima DF, Tanna DD (1989). Role of pulsed electromagnetic fields in recalcitrant nonunions. J Postgrad Med.

[123] Dunn AW, Rush GA (1984). Electrical stimulation in treatment of delayed union and nonunion of fractures and osteotomies. South Med J.

[124] Fontanesi G, Giancecchi F, Rotini R (1983). Treatment of delayed union and pseudoarthrosis by low frequency pulsing electromagnetic stimulation: study of 35 cases. Ital J Orthop Traumatol.

[125] Fontanesi JB, Dal Monte A, Rinaldi E (1984). The effect of low frequency pulsing electromagnetic fields for the treatment of congenital and acquired pseudarthroses. J. Bioelectricity.

[126] Freedman LS (1985). Pulsating electromagnetic fields in the treatment of delayed and nonunion of fractures: results from a district general hospital. Injury.

[127] Frykman GK, Taleisnik J, Peters G (1986). Treatment of nonunited scaphoid fractures by pulsed electromagnetic field and cast. J Hand Surg Am.

[128] Garland DE, Moses B, Salyer W (1991). Long-term follow-up of fracture nonunions treated with ICs. Contemp Orthop.

[129] Heckman JD, Ingram AJ, Loyd RD (1981). Nonunion treatment with pulsed electromagnetic fields. Clin Orthop Relat Res.

[130] Holmes GB (1994). Treatment of delayed unions and nonunions of the proximal fifth metatarsal with pulsed electromagnetic fields. Foot Ankle Int.

[131] Ito H, Shirai Y (2001). The efficacy of ununited tibial fracture treatment using pulsing electromagnetic fields: relation to biological activity on nonunion bone ends. J Nippon Med Sch.

[132] Massari L, Milena F, Ruggero C (2006). Biophysical stimulation with pulsed electromagnetic fields in osteonecrosis of the femoral head. J Bone Joint Surg Am.

[133] Madronero A, Pitillas I, Manso FJ (1988). Pulsed electromagnetic field treatment failure in radius non-united fracture healing. J Biomed Eng.

[134] Marcer M, Musatti G, Bassett CA (1984). Results of pulsed electromagnetic fields (ICs) in ununited fractures after external skeletal fixation. Clin Orthop Relat Res.

[135] Meskens MW, Stuyck JA, Feys H (1990). Treatment of nonunion using pulsed electromagnetic fields: a retrospective follow-up study. Acta Orthop Belg.

[136] Meskens MW, Stuyck JA, Mulier JC (1988). Treatment of delayed union and nonunion of the tibia by pulsed electromagnetic fields: a retrospective follow-up. Bull Hosp Jt Dis Orthop Inst.

[137] Saltzman C, Lightfoot A, Amendola A (2004). IC as treatment for delayed healing of foot and ankle arthrodesis. Foot Ankle Int.

[138] Satter SA, Islam MS, Rabbani KS (1999). Pulsed electromagnetic fields for the treatment of bone fractures. Bangladesh Med Res Counc Bull.

[139] Saxena A, Fullem B, Hannaford D (2000). Results of treatment of 22 navicular stress fractures and a new proposed radiographic classification system. J Foot Ankle Surg.

[140] Saxena A, DiDomenico LA, Widtfeldt A (2005). Implantable electrical bone stimulation for arthrodeses of the foot and ankle in high-risk patients: a multicenter study. J Foot Ankle Surg.

[141] Sharrard WJ, Sutcliffe ML, Robson MJ (1982). The treatment of fibrous nonunion of fractures by pulsing electromagnetic stimulation. J Bone Joint Surg Br.

[142] Bassett CA, Schink-Ascani M, Lewis SM (1989). Effects of pulsed electromagnetic fields on Steinberg ratings of femoral head osteonecrosis. Clin Orthop Relat Res.

[143] Bassett CA, Mitchell SN, Norton L (1978). Repair of nonunions by pulsing electromagnetic fields. Acta Orthop Belg.

[144] Das Sarkar S, Bassett CA (1991). Healing of nonunion of a fractured lateral condyle of the humerus by pulsing electromagnetic induction. Contemp Orthop.

[145] Gossling HR, Bernstein RA, Abbott J (1992). Treatment of ununited tibial fractures: a comparison of surgery and pulsed electromagnetic fields (IC). Orthopedics.

[146] Ito H, Shirai Y, Gembun Y (2000). A case of congenital pseudarthrosis of the tibia treated with pulsing electromagnetic fields: 17-year follow-up. J Nippon Med Sch.

[147] Akai M, Hayashi K (2002). Effect of electrical stimulation on musculoskeletal systems: a meta-analysis of controlled clinical trials. Bioelectromagnetics.

